# Exploring the Willingness and Understanding of Digital Rectal Examinations in Assessing Anorectal Conditions Among Saudi Patients in the Western Region of Saudi Arabia

**DOI:** 10.7759/cureus.67702

**Published:** 2024-08-24

**Authors:** Khalid A Basamih, Hanin M Alsaedi, Waleed K Alotaibi, Faeqah R Alharbi, Basem M Mufti, Jameel T Alrefai, Hassan H Meny, Ahmad O Bazarra, Mohammad M Alkot

**Affiliations:** 1 Department of Medicine and Surgery, College of Medicine, Umm Al-Qura University, Makkah, SAU; 2 Department of Medicine and Surgery, College of Medicine, Taibah University, Madinah, SAU; 3 Department of Community and Family Medicine, College of Medicine, Umm Al-Qura University, Makkah, SAU

**Keywords:** cross-sectional studies, knowledge, hemorrhoids, anorectal, digital rectal examination

## Abstract

Background: A digital rectal examination (DRE) is a crucial diagnostic examination used to identify various medical conditions by inserting a finger into the patient's rectum to check for abnormalities. Although clinically significant, DRE can be challenging, especially for first-time patients. Reasons for refusal often include misunderstandings about the examination, fear of the way the test is done, and feelings of embarrassment. This study seeks to explore the views, opinions, and perceptions of individuals in the Western region of Saudi Arabia towards DRE. The objective is to guide interventions and improve healthcare practices related to anorectal conditions in this population.

Method: In this cross-sectional study, we used a validated questionnaire, which was translated from English into Arabic, before distributing it to the target population. The target population included adults older than 18 years residing in the Western region of Saudi Arabia. Although our initial sample size was determined to be 385, we successfully recruited a larger sample of 1,147 participants. Data were statistically analyzed using IBM SPSS Statistics software for Windows, version 22 (IBM Corp., Armonk, NY).

Results: A total of 1,087 eligible participants completed the study questionnaire. Among them, 480 participants (44.2%) demonstrated a good overall awareness and understanding of DRE, while the majority, 607 (55.8%), had poor awareness. University-educated participants exhibited better awareness, with 363 (47.7%) showing good overall awareness compared to 103 (35.2%) of those with only secondary education, a statistically significant difference. Furthermore, 269 (46.7%) of students had a good awareness of DRE compared to 55 (34.8%) of unemployed participants. Notably, 218 (58.4%) of individuals working or studying in the medical field had a good awareness of DRE, compared to 207 (37.2%) in non-medical fields.

Conclusion: The majority of individuals showed limited knowledge regarding DRE. The findings suggest increasing public understanding and raising awareness of and importance of DRE for improving healthcare outcomes.

## Introduction

Digital rectal examination (DRE) is an invaluable diagnostic tool capable of uncovering crucial findings that can lead to the early detection and treatment of severe medical conditions, ultimately improving patient outcomes [1]. Its scope encompasses various medical areas, covering the management of conditions like rectal cancer, hemorrhoids, and fistulas, as well as the identification of bleeding such as melaena or hematochezia, assessment of fecal incontinence or constipation, evaluation of prostate cancer, and examination of pelvic floor prolapse or pelvic inflammatory disease. Moreover, DRE serves as a reliable method for evaluating anal tone in specific spinal, orthopedic, or neurological conditions, playing an essential role in the comprehensive assessment during secondary surveys for multi-trauma cases [2-4]. During a DRE, a healthcare professional inserts a finger into the rectum to assess any abnormalities or irregularities in the lower abdomen and pelvis. Typically, men are examined in a lateral position with knees bent upward towards the chest, although they may also be assessed while standing or partially bending forward at the waist. Women undergo an examination with their feet apart, raised in stirrups, or lying on their back with knees bent [5].

A DRE is a classical part of the examination in patients presenting with gastrointestinal bleeding. A retrospective cross-sectional study was conducted on 1,237 patients who presented to the emergency department with gastrointestinal bleeding. It showed that rectal examination has significantly decreased admission numbers, medical therapy, and endoscopy for patients with acute GI bleeding [6]. Despite its clinical significance, DRE poses substantial challenges, particularly for individuals undergoing the examination for the first time [7]. A study shows that refusal rates for DRE were higher among individuals without urological complaints (57%) and those anticipating pain or discomfort (18%), with attendees citing personal benefit (82%) and contribution to science (49%) as primary motives. Refusers tended to be older, less educated, with poorer general health, less knowledge about prostate cancer, and a less positive attitude towards screening compared to attendees [8]. Another study mentions other factors for refusing DRE, including misconceptions regarding prostate cancer screening, anticipation of severe discomfort, fear of potential cancer detection during the examination, and the perception of DRE as a source of shame [9].

Given the extensive global lack of research on this specific aim, a notable gap exists in the literature, particularly concerning the population of the Western region of Saudi Arabia. None have specifically addressed this topic within the population's unique cultural and societal context. Therefore, our study aims to bridge this critical gap. By investigating the attitudes, beliefs, and perceptions of the population towards DRE, we aspire to unearth invaluable insights that can inform targeted interventions and enhance healthcare practices for the assessment of anorectal conditions in this specific demographic. Through our research, we endeavor to contribute to advancing medical knowledge and optimizing healthcare delivery for the benefit of individuals in Makkah and beyond.

## Materials and methods

Study design

The current study is a cross-sectional descriptive online study. Data were collected by distributing an online questionnaire to the general population in the Western region of Saudi Arabia. The study secured ethical approval from the Biomedical Ethics Committee at the College of Medicine, Umm Al-Qura University (UQU), Makkah, Saudi Arabia (approval number: HAPO-02-K-012-2024-05-2153).

Study population

The study included the general population who lives in the Western region of Saudi Arabia, specifically adults over 18 years of age from both genders. The exclusion criteria consisted of individuals who were temporarily visiting the Western region of Saudi Arabia.

Study procedure

The targeted population in this study consists of adults over 18 years of age from the general population residing in the Western region of Saudi Arabia. According to the Saudi census of 2022, this population numbers 8,021,463 individuals [10]. We aimed to develop a questionnaire that is simple, concise, and easy for the population to understand. The questionnaire was adapted from a previously conducted study published in 2023 that assessed knowledge and willingness regarding DRE for assessing anorectal conditions in the Riyadh population [11]. An online Arabic questionnaire was created using Google Forms (Google Inc., Mountain View, CA). The respondents received electronic links along with information about the survey objectives, the target population, and a request to participate voluntarily. After obtaining approval from the UQU Institutional Research Board, the questionnaire was distributed electronically through data collectors to all eligible populations who met the inclusion criteria.

Data collection and management

The data were collected using a convenience sampling technique from the targeted population, with data quality ensured through supervision by data collectors. The collected data were directly transferred to a statistical database. This process was facilitated using an Arabic-language questionnaire, which included the following sections: consent form, sociodemographic data, and study questionnaire. To safeguard the confidentiality of participants' information, a system of codes, numbers, and pseudonyms was established. Access to the data was restricted solely to the researchers involved in the study.

Sample size determination

The Raosoft sample size calculator (Raosoft Inc., Seattle, WA) was used to determine the minimum sample size required for this study [12]. The population size of the Western region of Saudi Arabia is approximately 8,021,463. With a 95% confidence interval (CI), an anticipated frequency of 50%, and a design effect of one, the sample size was calculated to be 385 participants. To account for potential data loss, the total sample size was increased to 400 participants. We aimed to balance the sample size with 200 female and 200 male participants. 

Statistical analysis plan

After data extraction, it was revised, coded, and analyzed using IBM SPSS Statistics software for Windows, version 22 (IBM Corp., Armonk, NY). All statistical analyses were conducted using two-tailed tests, with a p-value of less than 0.05 considered statistically significant. Regarding awareness and perception of DRE, the overall score was obtained by summing all discrete item scores. Participants with an overall score of less than 60% of the maximum score were considered to have poor awareness levels, while those with a score of 60% or more were considered to have good awareness levels. Descriptive analysis based on frequency and percentage distribution was performed for all variables, including bio-demographic data, university affiliation, and medical and family history. Participants' knowledge and perceptions regarding DRE were tabulated, and their overall knowledge level was graphed. Cross-tabulation graphs were used to assess factors associated with participants' knowledge level about DRE, which was tested using Pearson's chi-square test and the exact probability test for small frequency distributions.

## Results

Out of 1,147 responses, a total of 1,087 participants who completed the study questionnaire were included. Participants ranged in age from 18 to 65 years, with a mean age of 23.8 ± 11.6 years. Of the respondents, 716 (65.9%) were female, 965 (88.8%) were Saudi nationals, and 761 (70%) had a university level of education. Exactly 634 (58.3%) were students, and 295 (27.1%) were employed. Regarding the study or work field, 373 (40.2%) were in the medical field. A total of 121 (11.1%) had anal and rectal diseases, and 36 (3.3%) had a relative with a rectal disease (Table 1).

**Table 1:** Biodemographic characteristics of study participants from the Western region of Saudi Arabia (n = 1,087)

Biodemographic data	Number	Percentage (%)
Age, in years		
18-25	738	67.9%
26-45	223	20.5%
46-65	126	11.6%
Gender		
Male	371	34.1%
Female	716	65.9%
Nationality		
Saudi	965	88.8%
Non-Saudi	122	11.2%
Educational level		
Below secondary	33	3.0%
Secondary	293	27.0%
University	761	70.0%
Employment		
Not employed	158	14.5%
Student	634	58.3%
Employed	295	27.1%
Study/employment field		
Non-medical	556	59.8%
Medical	373	40.2%
Have you ever suffered from anal and rectal diseases?		
My relative	36	3.3%
Myself	121	11.1%
No	930	85.6%

In terms of perception and awareness, 510 (46.9%) participants had heard of DRE, but only 71 (6.5%) had undergone the examination. Conditions that could be detected by a DRE, as reported by participants, included hemorrhoids (619, 56.9%), anal fissures (477, 43.9%), colorectal cancer (384, 35.3%), skin tags (321, 29.5%), and prostate cancer (316, 29.1%). A total of 478 (44%) participants agreed to undergo a DRE if recommended by their doctor. Among those who refused, the most reported reasons included feeling ashamed to be examined in front of the doctor (419, 56%), disgust at the way the test is done (361, 48.3%), fear of the examination method (328, 43.9%), and lack of symptoms (228, 30.5%). Exactly 171 (15.7%) agreed that anal and rectal examination was a painful and invasive procedure that should not be performed by doctors, while 611 (56.2%) agreed that customs and traditions constitute a barrier that causes delays or prevents people from undergoing a rectal examination. Only 80 (7.4%) agreed that if they or a family member had anal and rectal diseases, they would resort to traditional medicine. Additionally, 261 (24%) said they would agree to be examined if there was a screening clinic for anorectal diseases for prevention purposes using DRE (Table 2).

**Table 2:** Perception and awareness of study participants regarding digital rectal examination

Perception and awareness	Number	Percentage (%)
Have you ever heard of examining the anus and rectum with a finger?		
Yes	510	46.9%
No	577	53.1%
Have you ever had an anal and rectal examination with a finger?		
Yes	71	6.5%
No	1016	93.5%
Conditions can be detected by a digital rectal examination		
Hemorrhoids	619	56.9%
Colorectal cancer	384	35.3%
Skin tags	321	29.5%
Anal fissure	477	43.9%
Prostate cancer	316	29.1%
I don't know	326	30.0%
If your doctor recommended that you perform a digital rectal examination, would you agree?		
Yes	478	44.0%
No	609	56.0%
If no, what are the reasons for refusal?		
Feeling ashamed to be examined in front of the doctor	419	56.0%
I am disgusted by the way the test is done	361	48.3%
Fear of the method of conducting the examination	328	43.9%
Lack of symptoms	228	30.5%
Fear of the results of the examination	83	11.1%
None of the options mentioned	87	11.6%
Symptoms that will make you agree to undergo the examination		
Bleeding	609	56.0%
Pain	614	56.5%
Feeling of a lump in the anus and rectum	527	48.5%
changes in bowel movement (constipation, diarrhea)	192	17.7%
Nothing	209	19.2%
Anal and rectal examination is a painful and invasive procedure performed by a doctor and should not be performed by doctors		
Yes	171	15.7%
No	497	45.7%
I don't know	419	38.5%
Customs and traditions constitute a barrier to society that causes them to be late or prevent them from going to the doctor for a finger examination of the rectum		
Yes	611	56.2%
No	115	10.6%
Maybe	361	33.2%
If you or someone in your family was afflicted with anal and rectal diseases, would you resort to traditional medicine?		
Yes	80	7.4%
No	642	59.1%
Maybe	365	33.6%
If there was a screening clinic for anorectal diseases for the purpose of prevention using digital rectal examination, would you agree to be examined?		
Yes	261	24.0%
No	518	47.7%
Maybe	308	28.3%

Table 3 presents the perceptions and awareness of study participants about hemorrhoids. Regarding the definition of hemorrhoid, 535 (49.2%) chose dilated anal blood vessels, while 649 (59.7%) incorrectly chose anal injuries, and 403 (37.1%) said it is defined as pain in the area around the anus. Constipation and chronic diarrhea were the most reported causes of hemorrhoids (714, 65.7%), followed by a long period of sitting (649, 59.7%), dilation due to constant pressure during defecation (522, 48%), and using the bathroom for a long time (435, 40%). For the complications, 650 (59.8%) knew about hypertension, 595 (54.7%) knew about anemia, and 184 (16.9%) chose diabetes mellitus. Finally, preventive measures for hemorrhoids were also investigated, which showed that eating foods rich in fiber (853, 78.5%) was the most important one, followed by drinking plenty of fluids (776, 71.4%), defecation when needed (647, 59.5%), and avoiding fatty foods (479, 44.1%).

**Table 3:** Perception and awareness of the study participants regarding hemorrhoids

Hemorrhoid awareness	No	%
What is the definition of hemorrhoids?		
Itching	101	9.3%
Dilated anal blood vessels	535	49.2%
Pain in the area around the anus	403	37.1%
Anal injuries	649	59.7%
Fecal incontinence	241	22.2%
Bleeding	177	16.3%
Pain during urination	152	14.0%
Swelling in the area around the anus	404	37.2%
Ulceration in the area around the anus	295	27.1%
Urinary incontinence	73	6.7%
Diarrhea	82	7.5%
Abdominal pain	84	7.7%
Sweating	19	1.7%
Anal discharge	124	11.4%
Causes of hemorrhoids		
Dilatation due to constant pressure during defecation	522	48.0%
Constipation or chronic diarrhea	714	65.7%
Using the bathroom for a long time	435	40.0%
Sitting for a long time	649	59.7%
Not exercising	248	22.8%
Pregnancy	203	18.7%
Childbirth	252	23.2%
Others (vegetables, hot pathing, and contraceptive pills)	181	16.7%
Tight pants	168	15.5%
Complications of hemorrhoids		
Anemia	595	54.7%
Hypertension	650	59.8%
Diabetes	184	16.9%
Measures can be taken to prevent hemorrhoids		
Eat food rich in fiber	853	78.5%
Drink plenty of fluids	776	71.4%
Avoid fatty foods	479	44.1%
Defecate when needed	647	59.5%
Hot drinks	20	1.8%
Stop smoking	237	21.8%
Use tissues to clean	261	24.0%
Get enough sleep	282	25.9%
Others	296	27.2%

**Figure 1:**
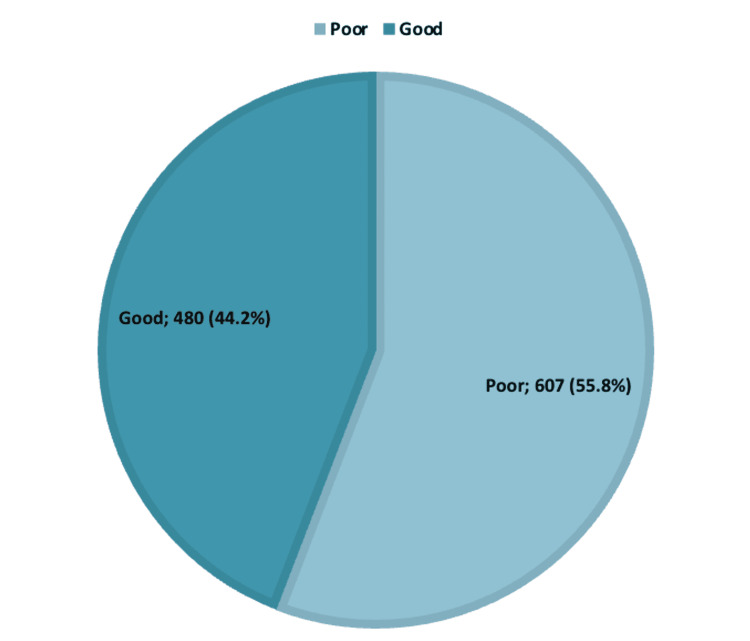
Participants' overall awareness and understanding of digital rectal examination

Regarding awareness and understanding of DRE, 480 (44.2%) of the study participants had good overall awareness and understanding, while the majority (607, 55.8%) had poor awareness (Figure 1).

Factors associated with participants' awareness and perception of DRE showed that 363 (47.7%) of university-educated participants had good overall awareness compared to 103 (35.2%) of those with only a secondary education, which was statistically significant (P = 0.001). Additionally, 296 (46.7%) of students had good awareness compared to 55 (34.8%) of unemployed participants (P = 0.001). Good awareness was found among 218 (58.4%) of medical field workers/students compared to 207 (37.2%) of the non-medical field group (P = 0.001) (Table 4).

**Table 4:** Factors associated with participants' awareness and perception of digital rectal examination from the Western region of Saudi Arabia P: Pearson's X^2^ test; *P < 0.05 (significant); ^: Exact probability test

Factors	Overall awareness and perception				p-value
	Poor		Good		
	No	%	No	%	
Age, in years					.540
18-25	406	55.0%	332	45.0%	
26-45	125	56.1%	98	43.9%	
46-65	76	60.3%	50	39.7%	
Gender					.683
Male	204	55.0%	167	45.0%	
Female	403	56.3%	313	43.7%	
Nationality					.346
Saudi	534	55.3%	431	44.7%	
Non-Saudi	73	59.8%	49	40.2%	
Educational level					.001*^
Below secondary	19	57.6%	14	42.4%	
Secondary	190	64.8%	103	35.2%	
University	398	52.3%	363	47.7%	
Employment					.026*
Not employed	103	65.2%	55	34.8%	
Student	338	53.3%	296	46.7%	
Employed	166	56.3%	129	43.7%	
Study / employment field					.001*
Non-medical	349	62.8%	207	37.2%	
Medical	155	41.6%	218	58.4%	
Have you ever suffered from anal and rectal diseases?					.913
My relative	21	58.3%	15	41.7%	
Myself	69	57.0%	52	43.0%	
No	517	55.6%	413	44.4%	

## Discussion

The study included 1,087 participants with a mean age of 23.8 years. Most of the participants were female Saudi nationals with a university level of education. Among them, only 373 (40.2%) were from the medical field. Awareness regarding DRE was moderate, with 510 (46.9%) having heard of it and 71 (6.5%) having undergone the examination. Reasons for refusal of the examination, if advised, included embarrassment, disgust, fear of the examination, and absence of symptoms. Some participants felt that the examination should not be performed by doctors and cultural and traditional barriers were cited as hindrances to seeking medical help. Additionally, misconceptions about hemorrhoids were identified, particularly regarding their causes and definitions. The results highlighted education level, student status, and medical occupation as significant predictors of awareness of DRE.

When asked about their awareness and experience with DRE, less than half of the participants reported having heard of DRE, and only 71 (6.5%) had undergone the examination. This aligns with related studies on the prevalence of acute gastrointestinal bleeding among patients presenting to the emergency department [6], suggesting low awareness and limited knowledge about this screening and diagnostic tool among the general population.

The respondents demonstrated a poor understanding of the types of diseases that can be diagnosed by DRE. Although more than half believed that hemorrhoids could be identified through DRE, fewer were aware of its application in detecting colorectal cancer, anal fissures, and prostate cancer. This underscores the significant need to raise public awareness about DRE and its numerous benefits.

Notably, 171 (15.7%) of respondents agreed that DRE was a painful and invasive examination that should not be performed by doctors. Furthermore, more than half believed that cultural and social taboos prevent individuals from seeking DRE. These perceptions highlight the necessity for substantial efforts in knowledge enhancement and promotion to address misconceptions and improve acceptance of DRE. Regarding hemorrhoids, the most recognized symptom was anal injuries, reported by 649 (59.7%) of participants, followed by dilated anal blood vessels, recognized by 535 (49.2%), and swelling in the area around the anus, reported by 404 (37.2%). Itching was the least recognized symptom, identified by only 101 (9.3%). The higher number of participants recognizing symptoms such as anal injuries and dilated blood vessels indicates a moderate level of health literacy on the basic symptoms of hemorrhoids. However, the low number of participants who reported itching as a symptom highlights the need for enhanced health literacy regarding other symptoms of hemorrhoids.

A study in Saudi Arabia indicates that there is limited awareness of hemorrhoid symptoms [13]. Awareness of hemorrhoid symptoms is essential, as it is an indication for performing DRE and facilitates acceptance and understanding from patients [14].

Many factors may affect the level of awareness and perception of DRE, including ethnicity and age. Both are important to investigate, as a study conducted in the United States shows that ethnicity and age significantly influence factors such as income and education, which, in turn, affect DRE acceptance. However, the study did not report a direct effect of age and ethnicity on DRE screening rates [15]. Ethnicity is a paradoxical factor that may show significance in some studies and not in others. For instance, one review found that Hispanic individuals are more likely to undergo DRE, while another study found no differences based on ethnicity [16], which aligns with our findings that nationality has no significant effect. Additionally, it is reported that people over 50 years old are more likely to undergo DRE [17], yet in our study, there is no significant effect of age on the level of awareness. Psychologically, fear has been shown to reduce acceptance and affect the perception of screening methods like DRE [15]. This is supported by another study indicating that fear of the examination is negatively associated with DRE acceptance. Regardless of the patient's education level, it is important to educate them about the examination and its importance.

Educating patients can reduce their fear and anxiety [16]. Sufficient education is essential to overcome this barrier and increase the acceptance level of medical examinations, as education and health are crucial to the overall well-being of individuals and communities [17].

In the current study, overall awareness and perception are significantly affected by educational level, employment, and the field of study or work. Interestingly, participants in high school show that 190 (64.8%) of them have poor knowledge, followed by participants with an educational level below secondary, with 19 (57.6%) having poor awareness levels. Additionally, a study indicates that having previous experience with anorectal diseases significantly improves one's perception and awareness of DRE [11]. However, our findings show no difference in awareness between individuals who have suffered from anorectal diseases and those who have not.

Regarding gender variation, our study found no significant differences in awareness and perception of DRE between males and females. Both genders exhibited high percentages of poor awareness, with 204 (55.0%) of males and 403 (56.3%) of females having low levels of awareness. It is also reported that both genders experience high levels of anxiety and fear before undergoing DRE [16].

Finally, to the best of our knowledge, this is the first study covering a large population in Saudi Arabia, and it adds to the literature, especially since there are not enough studies investigating the research topic. We collected a large amount from the Western region of Saudi Arabia; still, it is a limitation as the results cannot be generalized; further study is required to cover several populations for generalizability. A notable limitation is the unequal sex distribution, with more women responding to the survey than men. Another limitation is that the method of collection was an online survey; advanced collection methods in future studies will make the results more accurate. Also, we recommend some modifications to the questionnaire to investigate if the physician’s gender affects the feeling of fear by patients or not. Some modification is also recommended to assess if the feeling of embarrassment is affected by the gender of the patient. Lastly, as we discussed earlier, our findings suggest a poor level of awareness among the targeted population; therefore, we recommend that future researchers start to think about providing solutions for this issue and improving the level of awareness. 

## Conclusions

This study was conducted to explore the willingness and understanding of DRE in assessing anorectal conditions among Saudis in the Western region of Saudi Arabia. According to our findings, most people demonstrated poor awareness of DRE. This indicates a low level of awareness regarding the benefits and appropriate timing for performing DRE. Consequently, the Saudi Ministry of Health should focus on increasing public understanding of DRE and raising awareness about the dangers of neglecting this important examination, as well as the advantages it offers.
